# Supplementary material for: Matching sensor ontologies through siamese neural networks without using reference alignment
Source: PeerJ Comput Sci. 2021 Jun 18;7:e602. doi: 10.7717/peerj-cs.602 (PMC8237319; doi:10.7717/peerj-cs.602)
Supplement: Supplemental Information 1 [file peerj-cs-07-602-s001.zip › 301/backup/onto.html]

bibtex ontology


# bibtex ontology

Transformation of bibTeX into an OWL ontology

*Possible ontology to describe bibTeX entries.*  
Author: Nick Knouf <nknouf@mit.edu>  
Contributor:   
Date: 2003-12-29  
Version: 0.01

## Classes

**Entry** (Entry, *Base class for all entries)*
:   **Article** (Article, *An article from a journal or magazine.)*
    :   - #hasAuthor [1 +oo]
        - #hasTitle [1 +oo]
        - #hasJournal [1 +oo]
        - #hasYear [1 +oo]

    **Book** (Book, *A book with an explicit publisher.)*
    :   - #humanCreator [1 +oo]
        - #hasTitle [1 +oo]
        - #hasPublisher [1 +oo]
        - #hasYear [1 +oo]

    **Booklet** (Booklet, *A work that is printed and bound, but without a named publisher or sponsoring institution.)*
    :   - #hasTitle [1 +oo]

    **Conference** (Conference, *The same as INPROCEEDINGS, included for Scribe compatibility.)*


    **Inbook** (Inbook, *A part of a book, which may be a chapter (or section or whatever) and/or a range of pages. )*
    :   - #humanCreator [1 +oo]
        - #pageChapterData [1 +oo]
        - #hasTitle [1 +oo]
        - #hasPublisher [1 +oo]
        - #hasYear [1 +oo]

    **Incollection** (Incollection, *A part of a book having its own title.)*
    :   - #hasAuthor [1 +oo]
        - #hasTitle [1 +oo]
        - #hasBooktitle [1 +oo]
        - #hasPublisher [1 +oo]
        - #hasYear [1 +oo]

    **Inproceedings** (Inproceedings, *An article in a conference proceedings.)*
    :   - #hasAuthor [1 +oo]
        - #hasTitle [1 +oo]
        - #hasBooktitle [1 +oo]
        - #hasYear [1 +oo]

    **Manual** (Manual, *Technical documentation.)*
    :   - #hasTitle [1 +oo]

    **Mastersthesis** (Mastersthesis, *A Master's thesis.)*
    :   - #hasAuthor [1 +oo]
        - #hasTitle [1 +oo]
        - #hasSchool [1 +oo]
        - #hasYear [1 +oo]

    **Misc** (Misc, *Use this type when nothing else fits.)*


    **Phdthesis** (Phdthesis, *A PhD thesis.)*
    :   - #hasAuthor [1 +oo]
        - #hasTitle [1 +oo]
        - #hasSchool [1 +oo]
        - #hasYear [1 +oo]

    **Proceedings** (Proceedings, *The proceedings of a conference.)*
    :   - #hasTitle [1 +oo]
        - #hasYear [1 +oo]

    **TechReport** (TechReport, *A report published by a school or other institution, usually numbered within a series.)*
    :   - #hasAuthor [1 +oo]
        - #hasTitle [1 +oo]
        - #hasInstitution [1 +oo]
        - #hasYear [1 +oo]

    **Unpublished** (Unpublished, *A document having an author and title, but not formally published.)*
    :   - #hasAuthor [1 +oo]
        - #hasTitle [1 +oo]
        - #hasNote [1 +oo]

## Properties

**hasKey** #Entry -> http://www.w3.org/2001/XMLSchema#string *(The key for a particular bibTeX entry. Note that the rdf:ID for each Entry instance could be the bibTeX key as well, possibly making this property redundant.)*


**hasAddress** #Entry -> http://www.w3.org/2001/XMLSchema#string *(Usually the address of the publisher or other type of institution. For major publishing houses, van Leunen recommends omitting the information entirely. For small publishers, on the other hand, you can help the reader by giving the complete address.)*


**hasAnnotation** #Entry -> http://www.w3.org/2001/XMLSchema#string *(An annotation. It is not used by the standard bibliography styles, but may be used by others that produce an annotated bibliography.)*


**hasBooktitle** #Entry -> http://www.w3.org/2001/XMLSchema#string *(Title of a book, part of which is being cited. See the LaTeX book for how to type titles. For book entries, use the title field instead.)*


**hasCrossref** #Entry -> http://www.w3.org/2001/XMLSchema#string *(The database key of the entry being cross referenced.)*


**hasEdition** #Entry -> http://www.w3.org/2001/XMLSchema#string *(The edition of a book--for example, "Second". This should be an ordinal, and should have the first letter capitalized, as shown here; the standard styles convert to lower case when necessary.)*


**howPublished** #Entry -> http://www.w3.org/2001/XMLSchema#string *(How something strange has been published. The first word should be capitalized.)*


**hasInstitution** #Entry -> http://www.w3.org/2001/XMLSchema#string *(The sponsoring institution of a technical report.)*


**hasJournal** #Entry -> http://www.w3.org/2001/XMLSchema#string *(A journal name. Abbreviations are provided for many journals; see the Local Guide)*


**hasMonth** #Entry -> http://www.w3.org/2001/XMLSchema#string *(The month in which the work was published or, for an unpublished work, in which it was written. You should use the standard three-letter abbreviation, as described in Appendix B.1.3 of the LaTeX book.)*


**hasNote** #Entry -> http://www.w3.org/2001/XMLSchema#string *(Any additional information that can help the reader. The first word should be capitalized.)*


**hasNumber** #Entry -> http://www.w3.org/2001/XMLSchema#string *(The number of a journal, magazine, technical report, or of a work in a series. An issue of a journal or magazine is usually identified by its volume and number; the organization that issues a technical report usually gives it a number; and sometimes books are given numbers in a named series.)*


**hasOrganization** #Entry -> http://www.w3.org/2001/XMLSchema#string *(The organization that sponsors a conference or that publishes a manual.)*


**hasPublisher** #Entry -> http://www.w3.org/2001/XMLSchema#string *(The publisher's name.)*


**hasSchool** #Entry -> http://www.w3.org/2001/XMLSchema#string *(The name of the school where a thesis was written.)*


**hasSeries** #Entry -> http://www.w3.org/2001/XMLSchema#string *(The name of a series or set of books. When citing an entire book, the the title field gives its title and an optional series field gives the name of a series or multi-volume set in which the book is published.)*


**hasTitle** #Entry -> http://www.w3.org/2001/XMLSchema#string *(The work's title, typed as explained in the LaTeX book.)*


**hasType** #Entry -> http://www.w3.org/2001/XMLSchema#string *(The type of a technical report--for example, "Research Note".)*


**hasVolume** #Entry -> http://www.w3.org/2001/XMLSchema#nonNegativeInteger *(The volume of a journal or multivolume book.)*


**hasYear** #Entry -> http://www.w3.org/2001/XMLSchema#nonNegativeInteger *(The year of publication or, for an unpublished work, the year it was written. Generally it should consist of four numerals, such as 1984, although the standard styles can handle any year whose last four nonpunctuation characters are numerals, such as '(about 1984)'.)*


**hasAffiliation** #Entry -> http://www.w3.org/2001/XMLSchema#string *(The authors affiliation.)*


**hasAbstract** #Entry -> http://www.w3.org/2001/XMLSchema#string *(An abstract of the work.)*


**hasContents** #Entry -> http://www.w3.org/2001/XMLSchema#string *(A Table of Contents.)*


**hasCopyright** #Entry -> http://www.w3.org/2001/XMLSchema#string *(Copyright information.)*


**hasISBN** #Entry -> http://www.w3.org/2001/XMLSchema#string *(The International Standard Book Number.)*


**hasISSN** #Entry -> http://www.w3.org/2001/XMLSchema#string *(The International Standard Serial Number. Used to identify a journal.)*


**hasKeywords** #Entry -> http://www.w3.org/2001/XMLSchema#string *(Key words used for searching or possibly for annotation.)*


**hasLanguage** #Entry -> http://www.w3.org/2001/XMLSchema#string *(The language the document is in.)*


**hasLocation** #Entry -> http://www.w3.org/2001/XMLSchema#string *(A location associated with the entry, such as the city in which a conference took place.)*


**hasLCCN** #Entry -> http://www.w3.org/2001/XMLSchema#string *(The Library of Congress Call Number.)*


**hasMrnumber** #Entry -> http://www.w3.org/2001/XMLSchema#string *(The Mathematical Reviews number.)*


**hasPrice** #Entry -> http://www.w3.org/2001/XMLSchema#string *(The price of the document.)*


**hasSize** #Entry -> http://www.w3.org/2001/XMLSchema#string *(The physical dimensions of a work.)*


**hasURL** #Entry -> http://www.w3.org/2001/XMLSchema#string *(The WWW Universal Resource Locator that points to the item being referenced. This often is used for technical reports to point to the ftp or web site where the postscript source of the report is located.)*


**humanCreator** #Entry -> http://www.w3.org/2001/XMLSchema#string *(A generic human creator category, necessary in order to contain both author and editor.)*
:   **hasAuthor** #Entry -> http://www.w3.org/2001/XMLSchema#string *(The name(s) of the author(s), in the format described in the LaTeX book.)*


    **hasEditor** #Entry -> http://www.w3.org/2001/XMLSchema#string *(Name(s) of editor(s), typed as indicated in the LaTeX book. If there is also an author field, then the editor field gives the editor of the book or collection in which the reference appears.)*

**pageChapterData** #Entry -> \_ *(A generic property to hold page and/or chapter data.)*
:   **hasChapter** #Entry -> http://www.w3.org/2001/XMLSchema#nonNegativeInteger *(A chapter (or section or whatever) number.)*


    **hasPages** #Entry -> http://www.w3.org/2001/XMLSchema#string *(One or more page numbers or range of numbers, such as 42-111 or 7,41,73-97 or 43+ (the `+' in this last example indicates pages following that don't form a simple range). To make it easier to maintain Scribe-compatible databases, the standard styles convert a single dash (as in 7-33) to the double dash used in TeX to denote number ranges (as in 7-33).)*

## Individuals

<rdf:Description@ttp://purl.org/dc/elements/1.1/creator>
:   - rdf:type = <\_@http://www.w3.org/2002/07/owl#AnnotationProperty>
    - dc:title = 'this is a test title'

<rdf:Description@ttp://purl.org/dc/elements/1.1/description>
:   - rdf:type = <\_@http://www.w3.org/2002/07/owl#AnnotationProperty>

<rdf:Description@ttp://purl.org/dc/elements/1.1/date>
:   - rdf:type = <\_@http://www.w3.org/2002/07/owl#AnnotationProperty>

<rdf:Description@ttp://purl.org/dc/elements/1.1/subject>
:   - rdf:type = <\_@http://www.w3.org/2002/07/owl#AnnotationProperty>

<rdf:Description@ttp://purl.org/dc/elements/1.1/title>
:   - rdf:type = <\_@http://www.w3.org/2002/07/owl#AnnotationProperty>

<rdf:Description@ttp://purl.org/dc/elements/1.1/source>
:   - rdf:type = <\_@http://www.w3.org/2002/07/owl#AnnotationProperty>

<rdf:Description@ttp://purl.org/dc/elements/1.1/identifier>
:   - rdf:type = <\_@http://www.w3.org/2002/07/owl#AnnotationProperty>

<rdf:Description@ttp://purl.org/dc/terms/abstract>
:   - rdf:type = <\_@http://www.w3.org/2002/07/owl#AnnotationProperty>

<rdf:Description@ttp://purl.org/dc/terms/bibliographicCitation>
:   - rdf:type = <\_@http://www.w3.org/2002/07/owl#AnnotationProperty>

---

Generated by OWL2HTML
